# Vertical differentiation drives the changes in the main microflora and metabolites of carbon and nitrogen cycling in the early freeze–thaw period in the Qinghai Lake Basin

**DOI:** 10.3389/fmicb.2024.1329647

**Published:** 2024-04-08

**Authors:** Ni Zhang, Kelong Chen, Chenxi Wu, Hongchen Jiang, Yangong Du, Zhirong Chen, Xinye Wang, Desheng Qi, Ziwei Yang

**Affiliations:** ^1^Qinghai Province Key Laboratory of Physical Geography and Environmental Process, College of Geographical Science, Qinghai Normal University, Xining, China; ^2^Key Laboratory of Tibetan Plateau Land Surface Processes and Ecological Conservation (Ministry of Education), Qinghai Normal University, Xining, China; ^3^National Positioning Observation and Research Station of Qinghai Lake Wetland Ecosystem in Qinghai, National Forestry and Grassland Administration, Haibei, China; ^4^State Key Laboratory of Freshwater Ecology and Biotechnology, Institute of Hydrobiology, Chinese Academy of Sciences, Wuhan, China; ^5^State Key Laboratory of Biogeology and Environmental Geology, China University of Geosciences, Wuhan, China; ^6^Qinghai Provincial Key Laboratory of Geology and Environment of Salt Lakes, Qinghai Institute of Salt Lakes, Chinese Academy of Sciences, Xining, China; ^7^Qinghai Provincial Key Laboratory of Restoration Ecology for Cold Region, Northwest Institute of Plateau Biology, Chinese Academy of Sciences, Xining, China; ^8^College of Resources, Environment and Life Sciences, Ningxia Normal University, Guyuan, China

**Keywords:** Tibetan plateau, climate change, seasonal freeze–thaw alternations, microbial community and metabolic coupling, altitudinal gradients

## Abstract

Global climate change has altered the frequency of soil freeze–thaw cycles, but the response of soil microorganisms to different elevation gradients during the early freeze–thaw period remains unclear. So far, the influence of the altitudinal gradient on the microbial community and metabolic characteristics in the early freeze–thaw period of the Qinghai Lake Basin remains unclear. To this end, we collected soil at different elevations in the early freeze–thaw period of the Qinghai Lake Basin and investigated the influence of the elevation gradient on soil microbial community characteristics and soil metabolic processes as well as the corresponding environmental driving mechanism by high-throughput sequencing and LC–MS (Liquid Chromatograph-Mass Spectrometer) nontargeted metabolite determination. The results showed that Proteobacteria were the dominant microflora in the Qinghai Lake Basin. The dominant phyla associated with carbon and nitrogen are Proteobacteria and Firmicutes, both of which are significantly affected by elevation. The soil physicochemical factors jointly affected the soil microbial communities and metabolism. Total phosphorus nitrate nitrogen and pH were the main driving factors of the microbial community, and metabolites were sensitive to changes in chemical factors. In short, the microbial community structure and function, soil physicochemical factors and soil metabolic processes were significantly affected by the altitudinal gradient in the early freeze–thaw period, while the microbial community diversity showed no significant response to the altitudinal gradient. Additionally, a high potassium content in the soil may promote the growth and reproduction of bacteria associated with carbon and nitrogen cycling, as well as the production of metabolites.

## 1 Introduction

Soil microorganisms are important components and key driving factors of biogeochemical cycles (Bardgett and van der Putten, 2014; Thakur and Geisen, 2019), they are extremely sensitive to environmental changes (Shen et al., 2015; Deng et al., 2016), and their community characteristics have been used as key indicators of feedback climate warming (Davidson and Janssens, 2006). Freeze–thaw processes (periodic fluctuations in soil temperature and water phase) usually cause direct physical damage to soil microorganisms, and also change the ecological niche of soil microbial communities by affecting the soil microenvironment (Ren et al., 2018; Hou et al., 2020). Seasonal freeze–thaw soil has an abundance of microbial resources and rich soil nutrient content, with a remarkably wide distribution area (Wu et al., 2022). In recent decades, the impact of global climate change has led to the degradation of some permafrost into seasonally frozen ground. Previous research has consistently shown that soil microorganisms exhibit distinct responses to seasonal freeze–thaw cycles. For example, Liu et al. (2022) discovered that the increase in temperature as soil thaws stimulates the proliferation of numerous bacteria and lead to changes in the ecological network of these microorganisms. Chen et al. (2023) conducted a study on the soil bacterial community in alpine wetlands during seasonal freeze–thaw cycles. Their results showed that in the Alpha diversity of the soil bacterial community steadily decreased throughout the freeze–thaw period and the dominant bacterial community remained unchanged. Shi et al. (2023) investigated the effects of biochar and freeze–thaw cycles on bacterial communities and their diversity in cold black soil areas. The results showed that biochar significantly increased the richness and diversity of soil bacteria before and after the freeze–thaw. The seasonal freeze–thaw cycle typically includes three distinct phases. In the early phase of freeze–thaw cycle, the frozen soil layer is relatively thin and is subject to frequent freeze–thaw cycles. At the same time, temperature and moisture are subject to rapid fluctuations (Chen et al., 2023). The soil microenvironment is also changing rapidly, which has further effects on soil microorganisms. In addition, in the context of global climate change, the content of various soil carbon and nitrogen species in the soil may continue to increase during the initial freeze–thaw period (Liu et al., 2023). Therefore, it is imperative to study the characteristics of the soil microbial community during the initial freeze–thaw period.

As an important topographic factor, elevation change will cause changes in climate factors and soil physical and chemical properties, which will directly or indirectly lead to significant changes in biological and abiotic factors (He and Chen, 1997; Long et al., 2019) and further affect soil microorganisms (Zhou et al., 2021; Ma et al., 2022). With the rapid development of sequencing technology, there have been many studies on soil microbial changes at different elevations, and the research results have varied. Nottingham et al. (2018) showed that temperature was the main driving factor of soil microorganisms at different altitudinal gradients, and the richness of soil microbial species decreased with increasing elevation, while the difference in community composition increased with increasing elevation. Li et al. (2018) systematically studied the pattern of soil bacterial diversity along the elevational gradients of Gongga Mountain with an altitudinal range of 1800 ~ 4,100 m and found that soil bacterial diversity was distributed in a stepped pattern along the altitudinal gradient, and the change in bacterial diversity was not obvious at higher elevations (2,800 ~ 4,100 m). Shen et al. (2020) showed that bacteria and fungi had different diversity patterns along an elevational gradient. Bacterial diversity presented a U-shaped distribution, while fungal diversity decreased monotonically. Singh et al. (2014) studied the relationship between the bacterial diversity and community composition and elevation on Halla Mountain, Jeju Island, South Korea, and found that the bacterial community diversity and richness were correlated with the altitudinal gradient, which was mainly significantly affected by climate change. In summary, the regional differentiation of microorganisms is closely related to elevation, which is an important factor affecting the microbial community.

The temperature increase rate of the Qinghai-Tibet Plateau is significantly amplified with increasing elevation (Liu et al., 2009), which makes the freeze–thaw effect sensitive to climate change. The freeze–thaw pattern changes obviously with climate change (Borken, 2008), which further affects microbial community characteristics (Wang et al., 2013; Luo et al., 2014). While previous studies have provided valuable insights into the relationship between the freeze–thaw process or elevation and microbial community characteristics, there has been a lack of a comprehensive analysis combining the repeated freeze–thaw cycles with the vertical differentiation of microbial distribution patterns during the early phage of freeze–thaw cycle at high elevations. The Qinghai Lake Basin, located in the northeastern part of the Qinghai-Tibet Plateau, is ecologically sensitive and vulnerable to global climate change, which is crucial to maintaining the ecological balance of the region (Chen et al., 2009; Li et al., 2012). The basin is characterized by perennial low temperatures, long winters and short summers. Permafrost occurs mainly in the northwestern part of the region, while seasonal permafrost occurs in the central and eastern parts of the basin (Gao and Zhang, 2019).

Therefore, in this study, to explore the influence of different elevations on soil microorganisms in the early freeze–thaw period of the Qinghai Lake Basin, soil microorganisms at different elevations (3,200 ~ 4,100 m) in the early freeze–thaw period of the Qinghai Lake Basin were studied. The diversity index, community structure and functional groups of microbial communities under five altitudinal gradients were analyzed, and LC–MS untargeted metabolism was used to analyse the differential metabolites between groups and the corresponding KEGG enrichment pathway. Our objectives were to (1) study the dynamic changes in microbial communities and soil metabolites at different points along an altitudinal gradient in the early freezing–thawing period, (2) assess the response of microbial communities and soil metabolites to environmental factors under the influence of elevation gradients, and (3) analyse the interaction between microbial community changes and soil differential metabolites. The results can provide a theoretical basis for understanding the microbial mechanism of carbon and nitrogen cycling in the ecosystem of the Qinghai Lake Basin.

## 2 Materials and methods

### 2.1 Overview of the sampling sites

**Figure 1:**
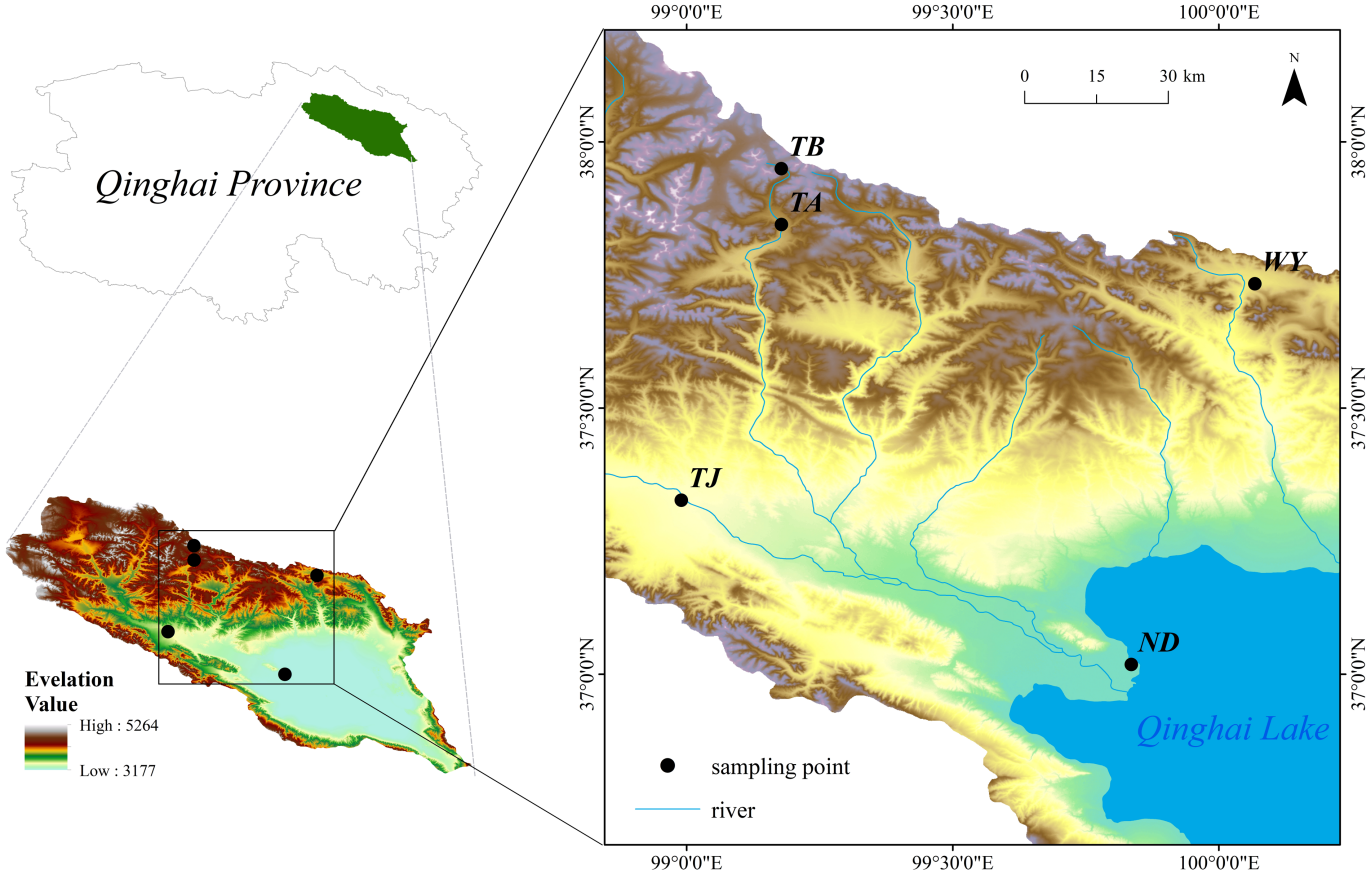
Sampling point distribution.

The Qinghai Lake basin (36°15′ ~ 38°20’N, 97°50′ ~ 101°20′E) is located on the northeastern Qinghai-Tibet Plateau at the intersection of the northwest arid area, eastern monsoon area and Qinghai-Tibet Plateau. Under climate warming, the land surface ecosystem of the basin, which is a typical plateau continental climate, changes dramatically. Surrounded by mountains, the basin is low in the southeast and high in the northwest, with an elevation between 3,194–5,174 m (Ren et al., 2020) and a drainage area of 29,661 km^2^ (Li et al., 2017). In this region, the diurnal temperature difference is large, the average annual temperature is −1.5 ~ 1.5°C, the precipitation is mainly concentrated in May–September, and the maximum rainfall is in July August. The distribution pattern of precipitation has obvious spatial and temporal heterogeneity, with the average annual precipitation ranging from 252 to 514 mm and the average annual evaporation ranging from 1,300 to 2,000 mm (Wang et al., 2022). The vegetation and soil types in the basin vary significantly with increasing elevation, and the vegetation types are divided into warm steppe, alpine steppe, alpine shrub and alpine meadow, and the soil types are chestnut soil, alpine meadow soil and alpine desert soil (Li et al., 2014). The distribution of sampling points is shown in Figure 1.

### 2.2 Experimental design, sample collection, and indoor processing

Five typical sample sites along Qinghai Lake and its tributaries were selected in combination with the elevation. Samples were collected in October 2021 at the early freezing–thawing stage of daily night soil freezing in the Qinghai Lake basin. The elevation interval of the sample points was approximately 200 meters, and they were named ND, TJ, WY, TA and TB in order of elevation (Supplementary Table S1). At each sampling site, five plots (1 m × 1 m) with flat terrain and uniform plant distribution were randomly selected. Five sampling points were randomly selected in each plot along an “S”-shaped curve. A soil auger with a diameter of 5 cm was used to collect topsoil samples from 0 to 10 cm. Each soil sample was evenly mixed, after which visible roots and other plant residues were removed, and then, the soil samples were passed through a 2-mm sieve and divided into two parts. One part was stored in a 10-mL Eppendorf tube and placed in a liquid nitrogen tank for high-throughput sequencing of soil-denitrifying microorganisms. The other part was stored at 4°C for the determination of soil-available potassium (AK), total potassium (TK), ammonium nitrogen (AN), nitrate nitrogen (NN), available phosphorus (AP), organic matter (OM), total nitrogen (TN), total carbon (TC), temperature (Tem), moisture (Moi), total phosphorus (TP) and pH.

AK and TK (flame photometry) were measured using an FP6410-flame photometer (Shanghai Instrument and Electronics Analytical GmbH, China). AN (sodium salicylate method) and NN (hydrazine sulfate reduction method) were measured using a continuous flow analyser (FUTURA, France). AP was measured using the double acid extraction - molybdenum antimony resistance colorimetric method (ultraviolet visible spectrophotometer UV-1900i, Shimadzu, Japan). OM was measured with the potassium dichromate-concentrated sulfuric acid external heating method (Plander Titrette Titrator, Germany). TN and TC were measured with an elemental analyser (Vario EL III, Elemental Analysis System GmbH, Germany). Temperature and moisture were measured with a TZS-1 K soil temperature and moisture meter (Zhejiang Top Technology Co., LTD, China). TP was measured using the sodium hydroxide melting - molybdenum-antimony resistance colorimetric method (ultraviolet visible spectrophotometer UV-1900i, Shimadzu, Japan). Soil pH (soil to water ratio 1:2.5) was measured with a pH meter (Mettler Toledo, Switzerland).

### 2.3 DNA extraction, polymerase chain reaction (PCR) amplification, and soil metabolomics

DNA was extracted from 0.25 g of homogenized soil sample, using a PowerSoil DNA Isolation Kit (MoBio Laboratories, Inc., Carlsbad, CA, USA) according to the manufacturer’s recommendation. To eliminate as much error as possible, DNA was extracted three times from each sample, mixed, and then subjected to 40 min of agarose gel electrophoresis at a 1% gel concentration. A NanoDrop UV–Vis spectrophotometer (ND-2000c, NanoDrop Technologies, Wilmington, DE, USA) was used for the determination of DNA purity and integrity. The PCR was performed with 16S rRNA-specific primers 341F (5’-CCTAYGGGRBGCASCAG-3′) and 806R (5’-GGACTACNNGGGTATCTAAT-3′) to amplify highly variable V3-V4 region sequences (Huse et al., 2008). The PCR process was performed using a 20 μL PCR system. The PCR instrument model was ABI GeneAmp® 9,700, and the specific PCR process was carried out as previously described (Tian et al., 2018). The PCR products were purified according to the standard of the AxyPrep PCR purification kit (AxyGen, San Francisco, CA, USA). Finally, amplicon sequencing was performed using the Illumina HiSeq platform from Shanghai Ling Biological Co., LTD. The total number of Circular Consensus Sequencing reads for 15 samples was 679,658.

### 2.4 Liquid chromatography–tandem mass spectrometry

Accurately weigh the correct amount of soil sample and place it into a 2 mL centrifuge tube. 600 μL of methanol–water was added (V:V = 7:3 with L-2-chlorophenylalanine, 4 μg/mL), steel ball was added and placed in the tissue grinder, grinding at 55 Hz for 90 s for 30 min at room temperature ultrasonicated, placed on ice for 30 min, centrifuged for 10 min at 12000 rpm and 4°C. The supernatant was filtered through a 0.22 μm membrane, and the filtrate was added to the test bottle for LC–MS detection. In addition, the quality control (QC) samples were prepared by uniformly mixing the extracts from each sample, and metabolites were extracted simultaneously with the experimental samples. LC/MS analyses were performed using the UHPLC-Q-Orbitrap HRMS® system (Thermo Fisher Scientific™, USA) and an ACQUITY UPLC BEH® C18 column (2.1 × 100 mm, 1.7 μm, Waters™, USA). In the positive ion mode, the mobile phase consists of 0.1% acetonitrile (B) and 0.1% formic acid water (A), and in negative ion mode, the mobile phase consists of acetonitrile (B) and 5 mM ammonium formate water (A). The gradient elution method was referred to the relevant literature (Li et al., 2022).

### 2.5 Sequencing and data analysis

Bioinformatics analysis of microbiome disembarkation data was conducted through the QIIME 2 (2019.4) process (Bolyen et al., 2019). Read labels and primers were removed from the original sequence data by the cutadapt plug-in (Martin, 2011), and then the DADA2 plug-in (Callahan et al., 2016) was used to obtain error-free biological sequences through quality control, error correction and chimaera removal for sequence noise reduction. The amplicon sequence variants (ASVs) obtained after deduplication were taken as the minimum taxa. Finally, taxonomic identification of each representative ASV sequence was conducted by the Ribosomal Database Project (RDP) classifier (Quast et al., 2012) based on the Silva Release138.1 (http://www.arb-silva.de) bacterial database (Wang et al., 2007). ASV numbers and annotation results were sorted through the Phyloseq package with R software (version 4.1.2).

The MicrobiotaProcess package was used to plot sample dilution curves and calculate alpha and beta diversity indices. The alpha diversity index was plotted by ggbox, PCA was plotted by ggordpoint, and *p* values between groups were calculated by the Wilcoxon rank sum test. ANOSIM results were calculated based on Bray–Curtis distance in the Vegan package, variance analysis with the aov function was performed to calculate p values of phylum level and genus level species abundance between different groups, and FAPROTAX (Liang et al., 2019) predicted the function of microflora. The LinkET package calculated the correlation and plotted the correlation network heatmap. After determining data in the Vegan package, redundancy analysis (RDA) was conducted based on a linear model to reveal the correlation among sample distribution, denitrification microbial community and environmental factors.

The mixOmics package was used to perform PLS-DA (Partial Least-Squares Discriminant Analysis), the ropls package was used to calculate VIp values between groups, and aov functions were used to calculate P values of metabolite abundance in different groups. The clusterProfiler package was used to count the number of metabolites in the KEGG pathway. Boxplots, species composition maps, differential species histograms, PLS-DA, metabolite expression calorigrams and enrichment pathway maps were all drawn by ggplot2. The psych package was used to calculate correlations between the data, and Cytoscape 3.9.1 was used to plot the network.

## 3 Results

### 3.1 Soil microbial diversity index at different elevations

**Figure 2:**
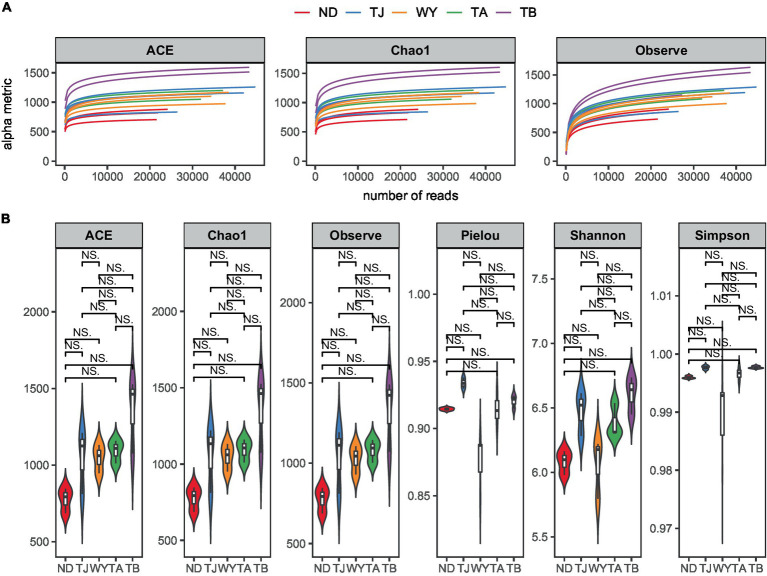
Soil microbial Alpha diversity at different elevations in the early freezing–thawing stage of the Qinghai Lake watershed. **(A)** Sample dilution curve; **(B)** Alpha diversity index.

**Figure 3:**
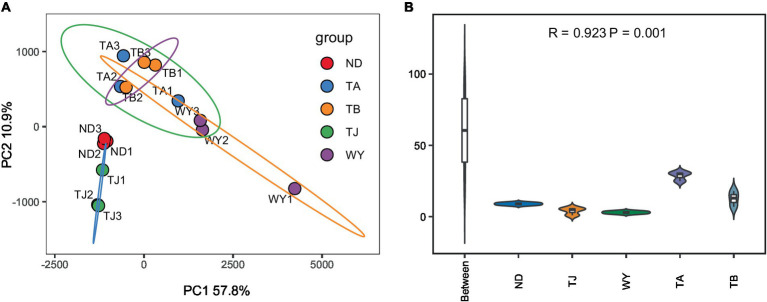
Soil microbial Beta diversity at different elevations in the early freezing–thawing stage of the Qinghai Lake watershed. **(A)** PCA principal component analysis; **(B)** Analysis of differences between ANOSIM groups.

To determine the response of the soil microbial community to the altitudinal gradient, the Illumina platform was used to conduct high-throughput sequencing. Figure 2A shows that the sequencing dilution curve of each sample tended to be flat, indicating that the amount of sequencing data was reasonable and that the current sequencing depth was sufficient to reflect the microbial community composition. In addition, the number of bacterial ASVs was the highest in the TB sample site and the lowest in the ND sample site. Alpha diversity analysis of soil microorganisms at different elevations in the early freeze–thaw period of the Qinghai Lake watershed (Figure 2B) showed that the species richness indices of microorganisms (ACE, Chao1, observed) showed an increasing trend with the increase in elevation. The Shannon index also showed the same rule. The Pielou index and Simpson index showed a U-shaped change with the increase in elevation, which first decreased and then increased, but the alpha diversity index showed no statistically significant difference among different groups (*p* > 0.05). The results of the PCoA (Figure 3A) showed that WY and TA had high soil heterogeneity, and the soil microbial community structure in the early freeze–thaw stage of the Qinghai Lake basin changed along the altitudinal gradient. The ANOSIM results (Figure 3B) further confirmed that this change was statistically significant (*p* = 0.001) and that grouping was significantly effective.

### 3.2 Soil microbial community structure at different elevations

**Figure 4:**
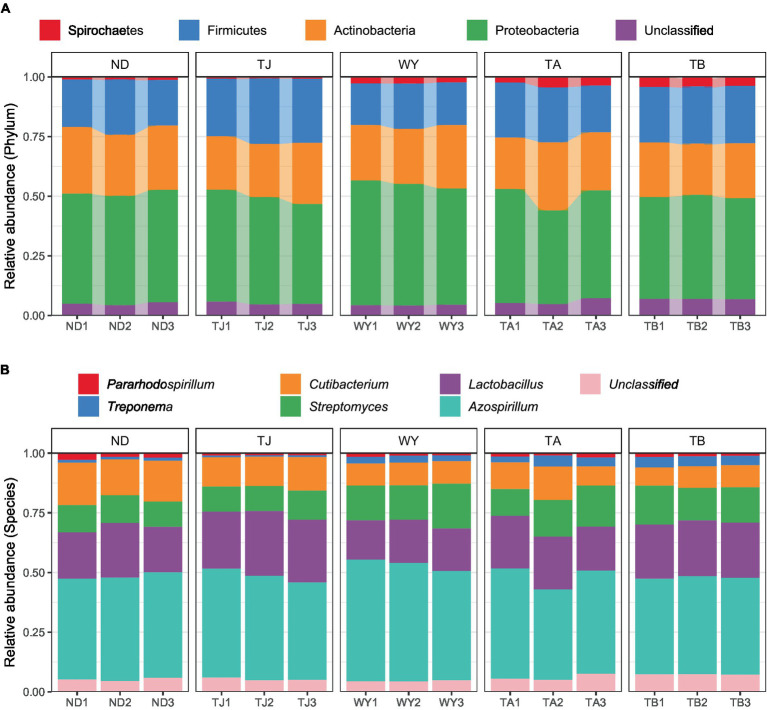
Composition of soil microbial species at different elevations in the early freeze–thaw period in the Qinghai Lake Basin. **(A)** Phylum level; **(B)** genus level.

**Figure 5:**
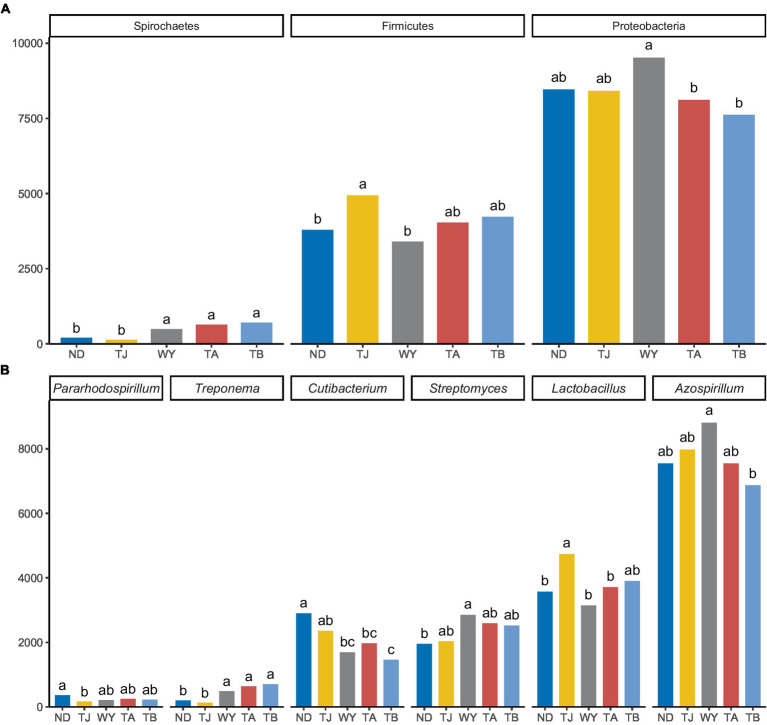
Different microflora at different elevations in the early freeze–thaw period in the Qinghai Lake Basin. **(A)** Phylum level differential species; **(B)** genus level differential species. abcd indicates a significant difference, the same letter indicates a nonsignificant difference, and different letters indicate a significant difference.

The soil microbial communities varied greatly at different elevations in the Qinghai Lake basin (Figure 4A). In general, Proteobacteria (43.99–50.64%), Actinobacteria (22.40–26.77%), Firmicutes (18.11–26.10%) and Spirochaetes (0.72–3.96%) were the four dominant phyla. With increasing elevation, the relative abundance of Spirochaetes showed an increasing trend (Figure 5A), and the relative abundance of Firmicutes in the TJ group was the highest. The variation trend of Proteobacteria with the altitudinal gradient showed that it first increased and then decreased.

At the genus level (Figure 4B), *Azospirillum* had the highest abundance, followed by *Lactobacillus* (16.71–24.80%), *Streptomyces* (10.63–15.20%), *Cutibacterium* (8.16–15.80%), *Treponema* (0.71–3.94%) and *Pararhodospirillum* (0.89–1.99%). Figure 5B shows that the relative abundance of *Pararhodospirillum* showed a U-shaped trend with increasing elevation, the relative abundance of *Treponema* and *Streptomyces* showed an increasing trend, and the relative abundance of *Cutibacterium* showed the opposite trend. The relative abundance of *Lactobacillus* in group TJ was significantly higher than that in other elevation gradients, and the relative abundance of *Azospirillum* showed a trend of first increasing and then decreasing with increasing elevation.

### 3.3 Functional groups of soil microorganisms

**Figure 6:**
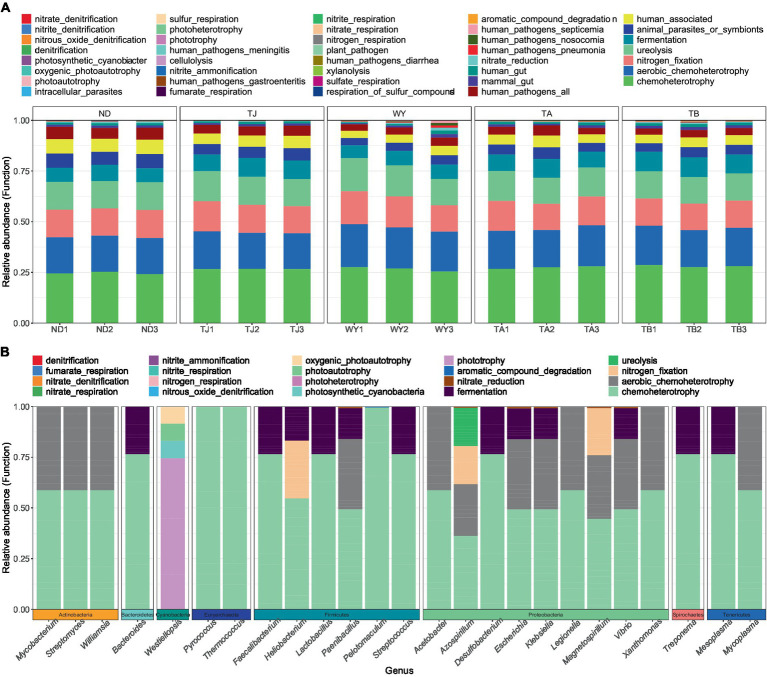
Functional groups of soil microorganisms at different elevations in the early freeze–thaw period in the Qinghai Lake Basin. **(A)** Distribution of functional groups of samples; **(B)** CN circulating functional group-related microflora.

**Figure 7:**
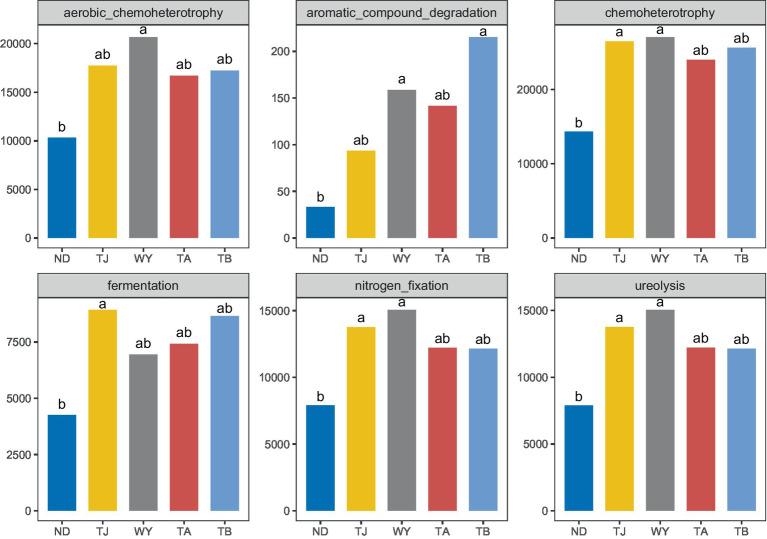
Differential functional groups of soil microorganisms at different elevations in the early freeze–thaw period in the Qinghai Lake Basin. abcd indicates a significant difference, the same letter indicates an insignificant difference, and different letters indicate a significant difference.

To clarify the functions of microbial communities under different altitudinal gradients, we predicted 38 functional groups of microbial communities in the early freeze–thaw stage of the Qinghai Lake basin through FAPROTAX (Figure 6A). The function of microflora was mainly chemoheterotrophy (24.64% ~ 28.07%), followed by aerobic chemoheterotrophy (17.80% ~ 20.35%), nitrogen fixation (13.32% ~ 14.86%), ureolysis (13.31% ~ 14.85%) and fermentation (6.84% ~ 9.54%). There were 20 functional groups related to the carbon-nitrogen cycle in the biogeochemical cycle (Figure 6B), and 25 genera-level microorganisms belonged to 8 phyla. Proteobacteria and Firmicutes were the main phyla. The main functional group of Westiellopsis was phototrophy, and chemoheterotrophy was the main functional group of other bacteria. *Azospirillum*, *Lactobacillus*, *Streptomyces* and *Treponema* had high relative abundances. Further analysis of the relationship between different allocational gradients and CN cyclic functional groups with relative abundances greater than 0.1% showed that 6 functional groups were significantly responsive to the allocational gradient (Figure 7), and the ND group had the lowest relative abundance of these functional groups. The relative abundance of aromatic_compound_degradation showed a gradual increase with elevation. The highest relative abundance in group TJ was significantly higher than that in group ND, and the remaining 4 functional groups showed a trend of first increasing and then decreasing with elevation.

### 3.4 Relationship among soil physical and chemical properties and microbial communities

**Figure 8:**
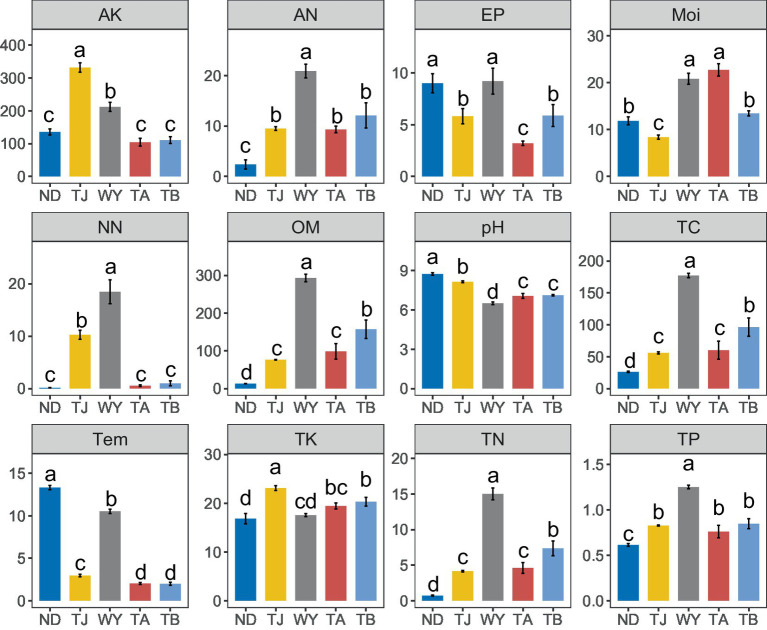
Analysis of Soil physicochemical factors at different elevations in the early freeze–thaw period in the Qinghai Lake Basin. AK, available potassium; AN, ammonium nitrogen; AP, available phosphorus; Moi, soil moisture; NN, nitrate nitrogen; OM, organic matter; pH, soil pH; TC, total carbon; Tem, soil temperature; TK, total potassium; TN, total nitrogen; TP, total phosphorus. abcd indicates a significant difference, the same letter indicates an insignificant difference, and different letters indicate a significant difference.

The physical and chemical properties of soil in the Qinghai Lake basin were significantly affected by the altitudinal gradient, and different soil environmental factors had different responses to different elevations (Figure 8). The AK and TK contents in the TJ group were significantly higher than those in the other groups. The pH and Tem values of the ND group were the highest among all groups. The AP content of the ND group was not significantly different from that of the WY group but was significantly higher than that of the other three groups. The Moi value of the TA group was the highest. In addition, the contents of NN, AN, OM, TC, TN, and TP in the WY group were the highest, and the contents of these 6 chemical factors were many times those in the other groups and showed a trend of first increasing and then decreasing with the gradual increase in elevation.

**Figure 9:**
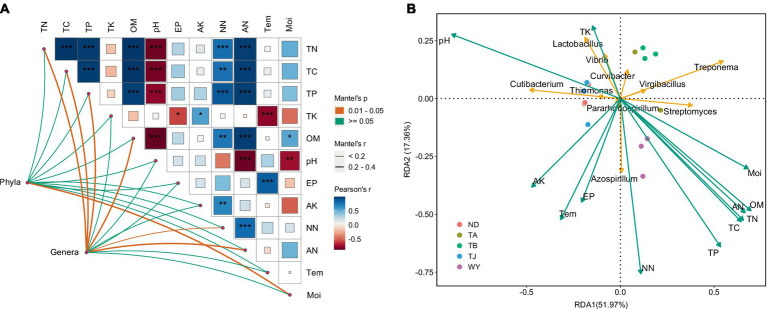
The relationship between soil physicochemical factors and dominant microflora at different elevations in the early freeze–thaw period in the Qinghai Lake Basin. **(A)** Correlation network diagram between dominant bacteria and soil physicochemical factors; **(B)** Redundancy analysis of horizontal dominant bacteria and soil physicochemical factors. AK, available potassium; AN, ammonium nitrogen; AP, available phosphorus; Moi, soil moisture; NN, nitrate nitrogen; OM, organic matter; pH, soil pH; TC, total carbon; Tem, soil temperature; TK, total potassium; TN, total nitrogen; TP, total phosphorus. abcd indicates a significant difference, the same letter indicates an insignificant difference, and different letters indicate a significant difference.

The results of the correlation analysis showed that there was a significant positive correlation between TN, TC, TP, OM, NN and AN (Figure 9A), a significant negative correlation between TN, TC, TP, OM, AN, Moi and pH, a significant positive correlation between AK and NN, AP and Tem, and a significant negative correlation between TK and Tem. Moi was significantly correlated with the dominant bacterial phyla but not with other physicochemical factors, and the dominant genera were significantly affected by TN, TC, TP, OM, NN, and AN. Furthermore, RDA showed that TP and NN were the most important influencing factors of dominant bacterial genera (Figure 9B). pH also plays an indispensable role in influencing the dominant microflora at the genus level. In addition, TN, TC, and TK also had a certain influence on the dominant microflora at the genus level.

### 3.5 Characteristics of soil microbial metabolism

**Figure 10:**
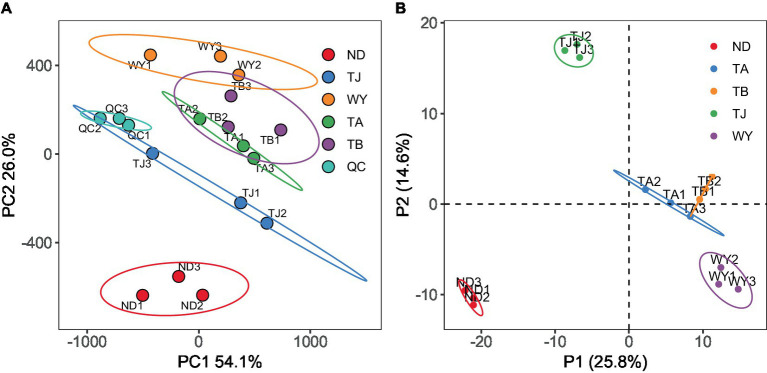
Metabolite characteristics of samples from different elevations of Qinghai Lake Basin during pre-freezing–thawing period. **(A)** Principal component analysis of samples. **(B)** Sample orthogonal partial least squares - discriminant analysis.

**Figure 11:**
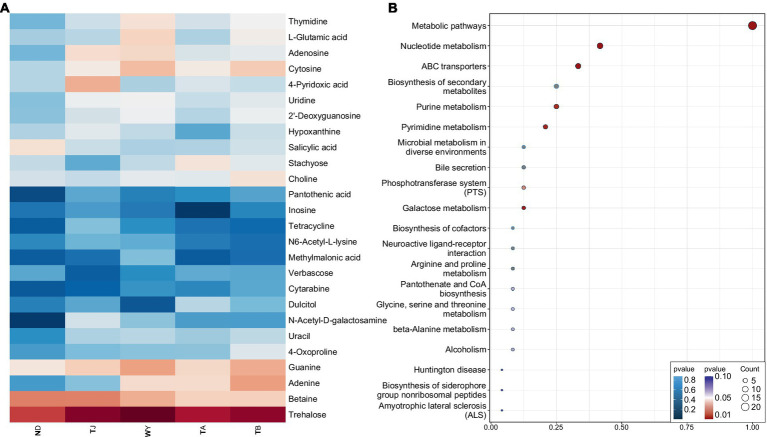
Expression and enrichment pathways of soil metabolites in different elevations of Qinghai Lake basin during pre-freezing–thawing period. **(A)** Differential metabolite expression calorigram. **(B)** Enriched bubble map of differential metabolite pathways.

To further explore the response mechanism of microorganisms at different elevations in the early freezing–thawing period, metabolomics analysis was performed using LC–MS. The PCA results showed that the QC samples were tightly clustered (Figure 10A), indicating the reproducibility of the experimental method and the stability of the instrumental analysis system. The data were stable and reliable and could be used for subsequent analysis. Partial least squares discriminant analysis (PLS-DA) was used to reveal the differences in metabolic profiles among the groups (Figure 10B). There were significant differences between the ND, TJ, and WY groups and the TA and TB groups, and the differences among the former groups were statistically significant, but the differences between the TA and TB groups were relatively small. With VIP greater than 1, absolute value of log2 (FC) greater than 1, P less than 0.05, and relative abundance greater than 0.1% as the screening conditions for metabolites, 26 qualified metabolites were found, and expression calorimetry maps were drawn (Figure 11A). The results showed that the relative abundance of most metabolites first increased and then decreased with the elevation gradient. The relative abundance of six metabolites (Inosine, N6-acetyl-L-lysine, tetracycline, 4-pyridoxic acid, hypoxanthine, and N-acetyl-D-galactosamine) was the highest in group TJ. In addition, the relative abundances of the 10 metabolites (2′-deoxyguanosine, adenosine, cytosine, uridine, L-glutamic acid, thymidine, guanine, methylmalonic acid, trehalose and uracil) were highest in the WY group. However, the relative abundance of dulcitol, verbascose and stachyose reached the maximum in the TA group. In addition, the relative abundance of 4-oxoproline, pantothenic acid, choline, adenine and cytarabine increased with the elevation gradient. Furthermore, the relative abundance of betaine and salicylic acid decreased gradually with increasing elevation.

To clarify the mechanism of the relationship between differential metabolites in biological processes, we performed KEGG pathway enrichment analysis on 26 different metabolites, and bubble maps were drawn based on the first 20 pathways of differential metabolite enrichment (Figure 11B). Except for verbascose and cytarabine, 24 metabolites were identified in these metabolic pathways. There were 10 different metabolites enriched in the nucleotide metabolism pathway. These metabolites were uridine, thymidine, guanine, cytosine, 2′-deoxyguanosine, adenine, adenosine, hypoxanthine, uracil and inosine. Eight metabolites were enriched in the ABC transporter pathway: trehalose, uridine, betaine, choline, 2′-deoxyguanosine, adenosine, L-glutamic acid and inosine. Purine metabolism and biosynthesis of secondary metabolites were enriched with 6 metabolites. The differential metabolites guanine, 2′-deoxyguanosine, adenine, adenosine, hypoxanthine and inosine were enriched in the former, and the differential metabolites enriched in the latter were trehalose, salicylic acid, tetracycline, adenine, L-glutamic acid and pantothenic acid. Five metabolites were enriched in the pyrimidine metabolism pathway, namely, uridine, methylmalonic acid, thymidine, cytosine and uracil. All of the above pathways were significantly enriched except biosynthesis of secondary metabolites. Moreover, five metabolites, uridine, 2′-deoxyguanosine, adenine, adenosine and inosine, play roles in multiple metabolic pathways. In the other pathways, only 1 ~ 3 different metabolites were enriched.

### 3.6 Correlation network analysis

**Figure 12:**
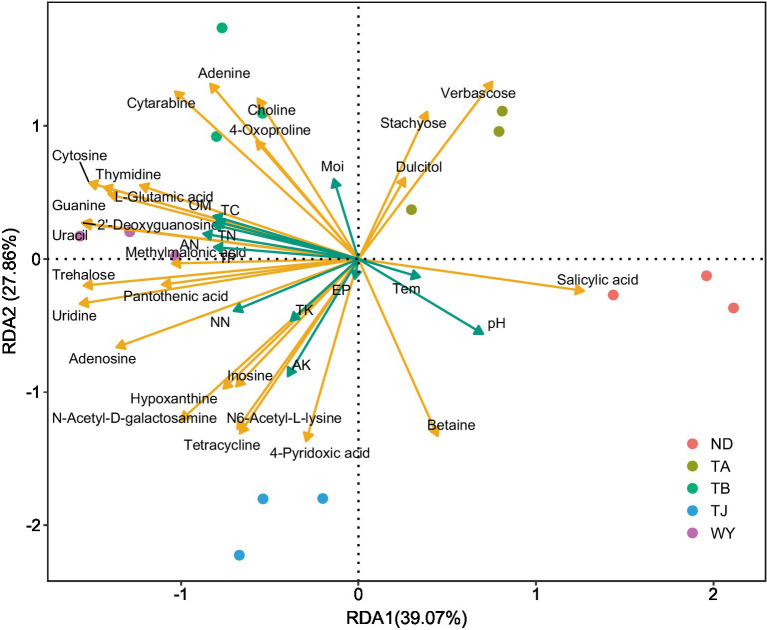
Redundancy analysis of soil differential metabolites and physicochemical factors in the Qinghai Lake Basin.

**Figure 13:**
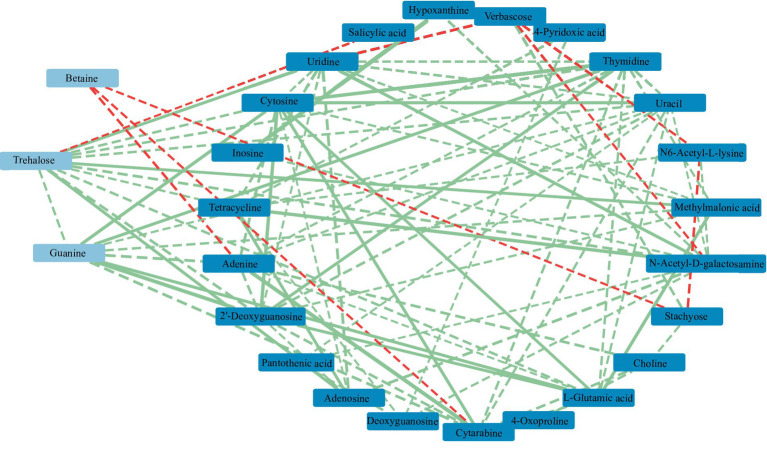
Correlation network of soil metabolites in the Qinghai Lake Basin. Metabolites with relative abundances greater than 1% are labeled in light blue, while those with relative abundances greater than 0.1% are labeled in dark blue. a correlation greater than 0.8 is a solid line, while a correlation less than 0.8 is a dotted line. The red segment indicates a negative correlation, while the green segment indicates a positive correlation. The thickness of the line represents the strength of the correlation weight; the stronger the correlation is, the thicker the line.

**Figure 14:**
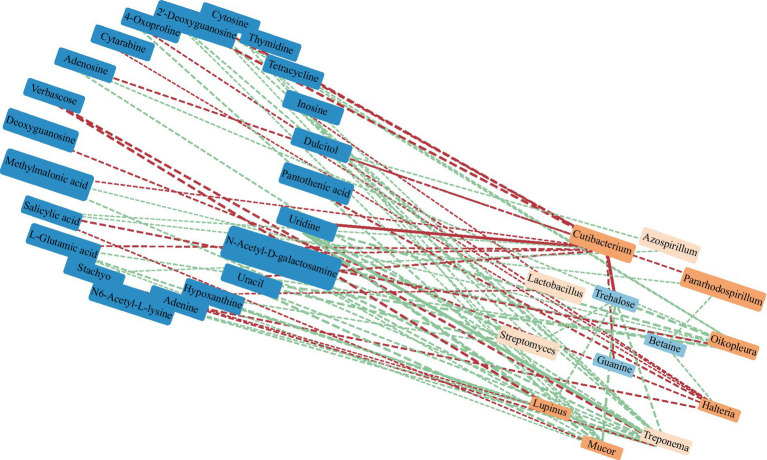
Correlation network between dominant soil bacteria at the genus level and differential metabolites in the Qinghai Lake Basin. Note: Metabolites with relative abundances greater than 0.1% are labeled in dark blue. The orange label is the dominant microflora at the genus level (Top 10); a correlation greater than 0.8 is a solid line, while a correlation less than 0.8 is a dotted line. The red segment indicates a negative correlation, while the green segment indicates a positive correlation. The thickness of the line represents the strength of the correlation weight; the stronger the correlation is, the thicker the line.

RDA found that AP had the least effect on different metabolites, and soil temperature, moisture (Tem, Moi) and TK had little effect on different metabolites (Figure 12). TC, TN, TP, AN, NN, OM and AK were positively correlated with the vast majority of different metabolites, and pH was negatively correlated with most of the different metabolites. Correlation network analysis of different metabolites clarified the relationship between metabolites (Figure 13), and most metabolites were positively correlated with each other (*r* > 0.6). In the three metabolites with relative abundances greater than 1% of trehalose, betaine and guanine, the correlation coefficient between trehalose and adenosine, methylmalonic acid, guanine and cytosine, thymidine, 2′-deoxyguanosine, and L-glutamic acid was greater than 0.8. In addition, there was a significant negative correlation between some metabolites (*r* < − 0.6). Betaine was negatively correlated with adenine, cytarabine and stachyose, trehalose was negatively correlated with salicylic acid, and verbascose was negatively correlated with N6-acetyl-lysine and N-acetyl-D-galactosamine. Furthermore, N6-acetyl-lysine was negatively correlated with stachyose, and the correlation coefficients among all the metabolites with negative correlations were less than 0.8. Moreover, 22 metabolites were associated with a significantly enriched bacterial group (|*r*| > 0.6) (Figure 14). Of the four genera associated with the CN cycle, *Azospirillum* was positively correlated with dulcitol and stachyose, *Lactobacillus* had a significantly negative correlation with inosine and hypoxanthine, and *Streptomyces* was positively correlated with trehalose, uridine, hypoxanthine, N-acetyl-D-galactosamine, inosine and uracil. *Treponema* was significantly associated with most of the differential metabolites; among them, uridine, N-acetyl-D-galactosamine, trehalose, L-glutamic acid, hypoxanthine, uracil, inosine, methylmalonic acid, cytosine, 2′-deoxyguanosine, adenosine, N6-acetyl-L-lysine and tetracycline were significantly positively correlated but significantly negatively correlated with verbascose and stachyose. Hypoxanthine, inosine, N-acetyl-D-galactosamine, uracil, uridine, trehalose and stachyose interact with two to three major bacterial communities of the carbon and nitrogen cycles, and they may play an important role in the carbon and nitrogen cycles of the soil ecosystem in the early freeze–thaw period of the Qinghai Lake Basin under the influence of an altitudinal gradient.

## 4 Discussion

### 4.1 Response of the microbial community to altitudinal gradients

Elevation can regulate the of the environment, affecting nutrients in the soil and thus influencing the diversity of bacterial communities (Shen et al., 2020). The diversity index of soil microorganisms in the Qinghai Lake Basin was less affected by the altitudinal gradient in the early freeze–thaw period, and the difference between groups was not statistically significant. The systematic investigation of a previous study on the pattern of soil bacterial diversity on Mount Gongga with an elevation gradient of 1800 ~ 4,100 m also showed that the variation in bacterial diversity was not obvious in the region with a higher elevation (2,800 ~ 4,100 m) (Li et al., 2018), which was consistent with the results of this study. Compared with other community characteristics of soil microorganisms, community composition is more sensitive to environmental changes (Papatheodorou et al., 2004). The changes in elevation did not affect the dominant bacterial species in the Qinghai Lake Basin (An et al., 2019; Li et al., 2019a), and Proteobacteria was still the most dominant bacteria at the phylum level. However, the relative abundance of Proteobacteria was significantly affected by the elevation gradient, and showed a trend of first increasing and then decreasing with the elevation gradient. Among the other dominant bacteria, the relative abundance of Actinobacteria did not change significantly. This stability is thought to be due to the microflora’s resistance to external pressures, their ability to absorb soil water and minerals, and also their promotion of the secretion of compounds that break down soil organic matter (Mackelprang et al., 2011; de Vries and Shade, 2013). Firmicutes can resist dehydration and extreme environments and enhance host metabolism (Zhao et al., 2018). Its relative abundance peaked at TJ and was significantly higher than that at ND and WY, which may be related to the high potassium content in TJ. The relative abundance of Spirochaetes was also significantly affected by elevation, and its relative abundance at WY was significantly higher than that at ND and TJ at lower elevations. In addition, the relative abundance of *Azospirillum*, as the dominant genus, first increased and then decreased with increasing elevation, and the change was significant. It is speculated that the microflora with significant changes in relative abundance may be due to their rapid response (Davidson and Janssens, 2006), which can adjust their state in time to cope with the changes in the soil microenvironment caused by changes in elevation.

The soil microbial community composition is closely related to its ecological functions (Wei et al., 2018). We predicted the microbial community function using FAPROTAX and found that the number of functional groups of microorganisms in the Qinghai Lake Basin was 38. Among them, 20 functional groups were involved in the carbon and nitrogen cycling processes of the biogeochemical cycle, and the corresponding 25 genus-level microbial communities were obtained through reverse screening data. They are characterized by chemoheterotrophy and belong to eight phyla, with *Proteobacteria* and *Firmicutes* being the main phyla. Wei et al. (2018) compared the characteristics and functions of bacterial communities in forest soil in China and showed that Proteobacteria, *Firmicutes* and *Actinobacteria* have heterotrophic functions and play important roles in carbon and nitrogen metabolism, elemental geochemical cycling and organic degradation. *Azospirillum*, *Lactobacillus*, *Streptomyces* and *Treponema* are abundant and may play an important role in soil carbon and nitrogen cycling in the early freeze–thaw period of the Qinghai Lake Basin. The six functional groups of carbon and nitrogen cycles were obviously responsive to the elevation gradient. The relative abundance of aromatic_compound_degradation gradually increased along the elevation gradient, and the relative abundance of the TJ group was the highest, which may indicate that high potassium content favors the degradation of aromatic compounds. The relative abundance of the remaining functional groups showed a trend of first increasing and then decreasing, which was consistent with the trend of change in the relative abundance of *Proteobacteria*. It may be that changes in the soil microenvironment caused by elevation differences affect the function of soil bacteria (Li et al., 2016; Zhou et al., 2016).

### 4.2 Interaction of physicochemical factors and microbial communities at different elevations

Soil environmental factors such as soil moisture and nutrient availability, play key roles in soil microbial community characteristics (Burns et al., 2013). Studies on soil environmental factors in the early freeze–thaw period of the Qinghai Lake Basin showed that there are differences in the ecological environment at different elevation gradations, and various indices of soil physiochemistry also change with elevation, but there was no unified rule. Temperature is an important factor affecting the soil microbial community (Zhou et al., 2016), and generally decreases steadily with increasing elevation. In this study, it was found that the soil temperature at the WY sampling site increased sharply and significantly, and the temperature changes at the other sampling sites were consistent with this rule, which may due to the comprehensive influence of the parent material of the soil formation and the regional microclimate. The Moi first decreased, then increased and then decreased along the elevation gradient, without a corresponding rule, which was consistent with the results of Tan et al. (2021). Among the other environmental factors, soil pH and AP showed U-shaped changes along the elevational gradient. The contents of AK, TK, NN, AN, OM, TC, TN, and TP initially increased and then decreased with the gradual increase in elevation. This trend could be due to the decrease in temperature caused by the increase in elevation, which in turn reduces the CO_2_ flux on the soil surface, thus increasing the accumulation of its content (Zhang et al., 2021). However, when soil temperature and moisture reach a certain value, it negatively affects the accumulation of soil chemical factors (Wang et al., 2018), resulting in a rapid reduction in their content.

As important indices for assessing soil nutrient cycling in ecosystems (Shen et al., 2015; Deng et al., 2016), soil microorganisms are significantly affected by soil physicochemical variables (Li et al., 2016; Bai et al., 2017). Microbial communities are influenced by several environmental factors, and the study of Li et al. (2016) on the Qinghai-Tibet Plateau also found that soil nutrient conditions such as total soil carbon, total organic carbon, total nitrogen, total phosphorus and total potassium significantly affected the soil microbial community. Previous studies have shown that the composition and function of the soil microbial community are mainly affected by soil moisture, pH, temperature, soil organic carbon and nutrients (Li et al., 2019b; Yang et al., 2020). Among them, soil pH can change the nutrient availability in the soil and thus influence the structure of the bacterial community structure (Zeng et al., 2016), which is considered an important factor affecting bacterial communities (Liu et al., 2020). In this study, TP and NN were found to be the most important factors affecting the soil microbial community, and pH, TN, TC, and TK also had important effects on the microbial community in this area, but soil temperature and moisture had less influence on the microbial community, which differed from previous research results. This may be due to the different optimal pH environments for microbial growth in different regions (Tripathi et al., 2018), leading to certain differences. Furthermore, Na et al. found that the bacterial community was not affected by soil moisture but by other soil characteristics (Na et al., 2019), which was consistent with the results of this study.

### 4.3 Soil metabolism is driven by environmental factors and interacts with microbial communities

Soil microbial metabolic diversity is closely related to soil nutrient cycling (Liang et al., 2016; Pang et al., 2019). The redundancy analysis revealed that the different metabolites were influenced by the interaction of environmental factors. Soil pH was significantly negatively correlated with most different metabolites. It is possible that the change in pH directly affects the enzyme activity of soil microorganisms and thus affects the metabolic process of soil microorganisms (Zhang et al., 2015). TC, TN, TP, AN, NN, OM, and AK were positively correlated with most different metabolites, indicating that sufficient nutrient availability and relative balance of soil nutrients could promote microbial metabolic processes (Cui et al., 2019). Most of the different metabolic pathways were enriched in pyrimidine metabolism, purine metabolism, biosynthesis of secondary metabolites, ABC transporters and nucleotides. Most metabolites in the five metabolic pathways also showed a significant positive correlation. This may be due to the allelopathy caused by microbial interaction, which promotes the metabolic process and results in most changes in metabolite content tending to be consistent. This study found that 22 metabolites were significantly correlated with dominant bacteria at the genus level, and previous studies also confirmed that the relative abundance of these groups was closely related to functional genes of metabolic pathways such as carbohydrate metabolism, energy metabolism, amino acid metabolism and environmental adaptation (de Scally et al., 2016). In addition, *Virgibacillus* showed a significant negative correlation with verbascose and stachyose, which may be related to the use of sugars by *Virgibacillus* acid production (Heyndrickx et al., 1950). *Lactobacillus* has a significant negative correlation with inosine and hypoxanthine, indicating that *Lactobacillus* has an inhibitory effect on purine metabolism (Wu et al., 2021), and this change is likely related to the maintenance of acid–base balance in the environment.

## 5 Conclusion

The species richness and Shannon index indices of microorganisms showed an increasing trend with increasing elevation. The Pielou index and Simpson index showed a U-shaped change with increasing elevation. Proteobacteria were the main microbiota associated with carbon and nitrogen cycle, and the trend of relative abundance was consistent with that of carbon and nitrogen content. Chemoheterotrophy was the main functional group of microorganisms associated with the carbon and nitrogen cycle. There were 26 different metabolites at different elevations, most of which were enriched in five metabolic pathways of KEGG, showing a significant positive correlation. Lactobacillus has an inhibitory effect on purine metabolism. Total phosphorus and nitrate nitrogen are the most important factors affecting microbial communities, and soil metabolites were mainly affected by soil chemical factors. *Azospirillum*, *Lactobacillus*, *Streptomyces*, and *Treponema* were the most dominant general-level microbial communities associated with the carbon and nitrogen cycling, and they were significantly positively correlated with most metabolites.

## Data availability statement

The datasets presented in this study can be found in online repositories. The names of the repository/repositories and accession number(s) can be found at: https://www.ncbi.nlm.nih.gov/, PRJNA984019.

## Author contributions

NZ: Conceptualization, Data curation, Investigation, Writing – original draft. KC: Conceptualization, Writing – review & editing. CW: Conceptualization, Writing – review & editing. HJ: Data curation, Writing – review & editing. YD: Data curation, Writing – review & editing. ZC: Investigation, Writing – review & editing. XW: Investigation, Writing – review & editing. DQ: Software, Writing – review & editing. ZY: Software, Writing – review & editing.
