# Supplementary material for: Vertical differentiation drives the changes in the main microflora and metabolites of carbon and nitrogen cycling in the early freeze–thaw period in the Qinghai Lake Basin
Source: Front Microbiol. 2024 Apr 8;15:1329647. doi: 10.3389/fmicb.2024.1329647 (PMC11033449; doi:10.3389/fmicb.2024.1329647)
Supplement: Supplementary file 1 [file Table_1.DOCX]

Table S1 Sample point information

| Sample sites | Longitude | Latitude | Altitude |
| --- | --- | --- | --- |
| ND | 36°58′50″ E | 99°52′47″ N | 3250 m |
| TJ | 37°19′37″ E | 98°59′24″ N | 3490 m |
| WY | 37°43′60″ E | 100°4′4″ N | 3720 m |
| TA | 37°50′41″ E | 99°10′42″ N | 3910 m |
| TB | 37°56′58″ E | 99°10′41″ N | 4105 m |
